# Origin Detection During Food-borne Disease Outbreaks - A Case Study of the 2011 EHEC/HUS Outbreak in Germany

**DOI:** 10.1371/currents.outbreaks.f3fdeb08c5b9de7c09ed9cbcef5f01f2

**Published:** 2014-04-01

**Authors:** Juliane Manitz, Thomas Kneib, Martin Schlather, Dirk Helbing, Dirk Brockmann

**Affiliations:** Department of Statistics and Econometrics, University of Göttingen, Göttingen, Germany; Department of Statistics and Econometrics, University of Göttingen, Göttingen, Germany; School of Business Informatics and Mathematics, University of Mannheim, Mannheim, Germany; Swiss Federal Institute of Technology, ETH Zurich, Zurich, Switzerland; Risk Center, ETH Zurich, Zurich, Switzerland; Northwestern Institute on Complex Systems, Northwestern University, Evanston, IL, United States of America; Northwestern Institute on Complex Systems, Northwestern University, Evanston, IL, United States of America; Robert Koch Institute, Berlin, Germany

## Abstract

The key challenge during food-borne disease outbreaks, e.g. the 2011 EHEC/HUS outbreak in Germany, is the design of efficient mitigation strategies based on a timely identification of the outbreak's spatial origin. Standard public health procedures typically use case-control studies and tracings along food shipping chains. These methods are time-consuming and suffer from biased data collected slowly in patient interviews. Here we apply a recently developed, network-theoretical method to identify the spatial origin of food-borne disease outbreaks. Thereby, the network captures the transportation routes of contaminated foods. The technique only requires spatial information on case reports regularly collected by public health institutions and a model for the underlying food distribution network. The approach is based on the idea of replacing the conventional geographic distance with an effective distance that is derived from the topological structure of the underlying food distribution network. We show that this approach can efficiently identify most probable epicenters of food-borne disease outbreaks. We assess and discuss the method in the context of the 2011 EHEC epidemic. Based on plausible assumptions on the structure of the national food distribution network, the approach can correctly localize the origin of the 2011 German EHEC/HUS outbreak.

## Introduction

Due to intensified mass production, facilitated world-wide shipping and novel food manufacturing methods, food-borne disease outbreaks occur more frequently with increasing impacts on society, public health institutions, the economy, and food industry^1^. An estimated 60% of annual gastrointestinal illnesses for each adult in the general population of the United States is caused by food-borne diseases^2^. Moreover, diarrhoea is the second leading cause of morbidity and mortality among children under five years worldwide^3^. food-borne diseases impose enormous financial burden on health care services, routine surveillance and public health investigations, and trigger substantial productivity impacts and product recalls by the food industry. For seven food-borne pathogens an annual burden of $6.5-$34.5 billion in the United States alone was estimated^4^.

One of the most substantial challenges in this context is determining the spatial origin of the contaminated food vehicle, which causes the epidemic, for earlier and more effective disease containment. Several factors make detection of the food-borne disease outbreak origin challenging, e.g. population growth, changing eating habits, globalization of food supply chains, production and processing innovations, and microbiological adaptation^1^^,^ 5. Furthermore, public health institutes have limited resources to solve issues such as underreporting and low specificity in the association between aetiology and food vehicle^6^. Origin reconstruction is a complex problem because the effects of contaminated food typically occur with a significant time lag and incidence patterns are geographically incoherent. Additionally, specific transport pathways are generally not monitored. More importantly, food distribution networks are multi-scale, spanning length-scale of hundreds to thousands of kilometers, delivering to and within spatially heterogeneous populations. Consequently, it is generically impossible to estimate the geographic origin of the phenomenon based on geometric aspects of the spatial distribution of reported cases. Only for 66% of the outbreaks, public health investigations identified evidence concerning the infection source^7^.

These practical difficulties were particularly striking during the German 2011 EHEC (enterohemorrhagic *Escherichia coli*) outbreak, which affected 3,842 people with unusually high rates of severe HUS (hemolytic-uremic syndrome) cases and mortality. The EHEC/HUS outbreak raised the awareness of timely and efficient origin reconstruction methods and their importance to society, public health institutions, risk assessment authorities and the food industry 2. There is no general procedure for food-borne disease outbreak investigations, that fits a particular event perfectly. However, the World Health Organization (WHO)^8^ provides practical standard guidelines for the investigation and control of food-borne disease outbreaks as a multi-disciplinary task which requires information from many sources.First, an unusual accumulation of disease reports has to be detected and defined as an outbreak. After pathogen specification, initial cases are investigated with regard to common factors and clinical and food specimens are sampled. The corresponding microbiological 'fingerprinting' of strains may also identify case relatedness and/or potential sources of contamination.From associated food and environmental samples, backward tracings are initiated to determine the origin. Furthermore, a case definition can be established to identify outbreak related cases and to collect their information on a standardized questionnaire.Using this data, analytical investigations, such as case-control and cohort studies, are performed to test hypotheses about the transmission vehicle and origin. The outbreak source is determined by combining all collected information, otherwise further analytical studies are required.Finally, the potential origin and transmission routes are controlled using forward tracings from contamination to the outbreak cases. Several attempts to improve traceability of food products to their geographical origin have been developed including technical innovations^9^, microbiological advances^10^, or food forensics^11^. However, detection of outbreak origin remains time-consuming and cost-intensive.

Network theory and network models have become the most important tools for understanding and predicting epidemics in general^12^^,^ 13 ^,^ 14. The majority of studies focuses on spatial disease dynamic systems in which networks quantify the coupling strength or transportation fluxes between spatially distributed populations. Almost all studies aim at understanding and forecasting the future time course of an epidemic based on the topological connectivity of the underlying transport networks^15^^,^ 16. Furthermore, most studies focus on human-to-human transmissible diseases. Little work has been done, however, on the inverse problem, also known as the 'zero patient' problem in epidemics. Shah and Zaman 17 ^,^ 18 developed a universal source detection maximum likelihood estimate, which assumes virus spread in a general graph along a breadth-first-search tree and derive theoretical thresholds for the detection probability. Pinto at al. 19 extended this estimate for partially observed transmission trees. Alternative origin reconstruction methods are based on shortest paths or consequent diameter from transmission trees 20 ^,^ 21. Prakash et al. 22 and Fioriti and Chinnici 23 developed methods based on spectral techniques to identify a (set of) origin nodes on a transmission network. They utilize a close relationship of source estimation and node centrality as shown by Comin and da Fontoura Costa 24. However, these methods require comprehensive knowledge of the transmission network, which is rarely the case.

Here we apply a recently developed network-geometric approach for epicenter reconstruction^25^ to food-borne diseases. This approach is based on a plausible redefinition of spatial separation and the introduction of an effective distance derived from the underlying food distribution network in combination with viewing the contagion process from the perspective of a specific node in the network. Using the effective distance method, complex spreading patterns can be mapped onto simple, regular wave propagation patterns if and only if the actual outbreak origin is chosen as the reference node. This way, the method can determine the correct outbreak origin based on the degree of regularity of the measured prevalence distribution when viewed in the effective distance perspective. This reconstruction is successful without the knowledge of the detailed infection hierarchy. Here, the underlying network captures the underlying transportation of the contaminated food rather than the mobility pattern of humans.

## German EHEC O104:H4/HUS outbreak 2011

Regarding the number of severe HUS cases, the 2011 EHEC/HUS outbreak in Germany, has been the largest *E. coli* outbreak reported worldwide. Between May 2 and July 26, 2011, 3,842 outbreak associated EHEC cases were reported to the Robert Koch-Institute (RKI), the German Federal Public Health and Surveillance Institute. This included 855 severe HUS cases (22.3%) and 53 patients (1.4%) died. The outbreak was caused by a rare serotype O104:H4 which infected predominantly adults (median age, 43 years), particularly women (68%), and resulted in high HUS and mortality rates 26. In the previous years, between 925 and 1,283 cases were reported annually, mostly in children. The majority of the infection cases was observed in Northern Germany, which resulted in a higher incidence (number of cases per 100,000 inhabitants) for the corresponding districts than the overall one for Germany (see Fig. 1). Extensive investigations were conducted by the Task Force EHEC, which included a matched case-control study, a recipe-based restaurant cohort study, and backward-/forward-tracings 27. The entire process was complicated, resource demanding and time-consuming. All investigations required a large amount of data that are typically biased, incomplete, erroneous, and sometimes contradictory. The tracings require a large amount of trained personnel and their success depends on the results of epidemiological studies.Only the combination of several study designs finally lead to the determination of sprouts as the transmission vehicle and the identification of their origin, a farm in Bienenbüttel located in the district Uelzen, Lower Saxony. On June 10, 38 days after outbreak onset, the public was informed to avoid sprout consumption and the responsible production farm was closed.

**E. coli incidence in Germany during 2011 EHEC/HUS outbreak.**
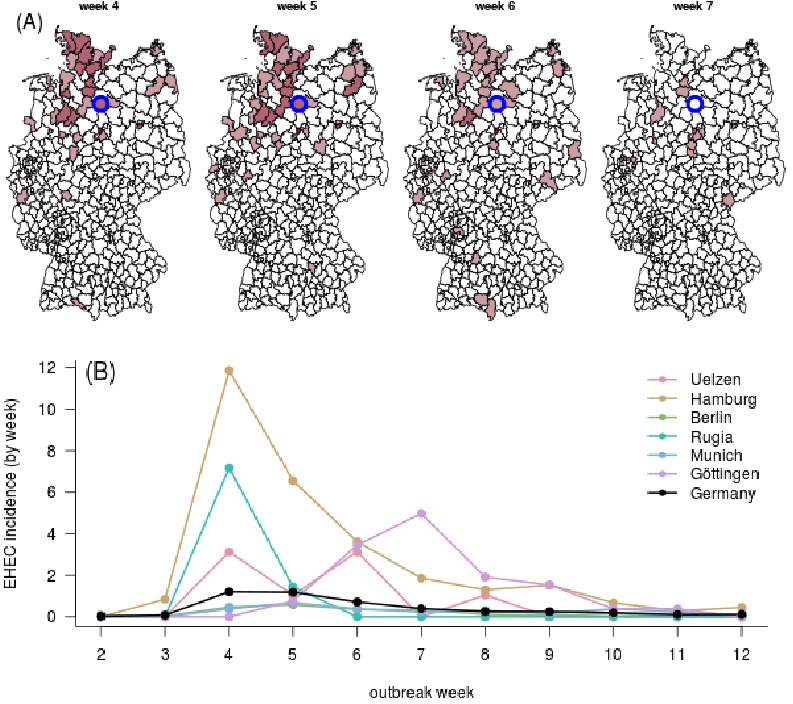
**(A)** Each panel depicts a different outbreak week (May 30th until June 20th, 2011). Color intensity quantifies infection counts in for each of the German districts (Data source: 28, Map source: 29). The alleged origin of outbreak (district Uelzen) is marked in blue.** (B)** Time course of E. coli incidence for selected districts. For reference, the overall German incidence per district is shown in black.

The severe impact of the disease on the population and industry, the fast and wide spread due to mass production and optimized food shipping, and the large public attention emphasize the need for fast and efficient outbreak origin localization.

## Network-theoretic origin detection

We consider a model network for spatial food distribution, where nodes \begin{equation*}\small{m=1,\ldots,M=412}\end{equation*} represent administrative districts in Germany. Links \begin{equation*}\small{F_{nm}}\end{equation*} quantify the amount of goods that are shipped from node \begin{equation*}\small{m}\end{equation*} to \begin{equation*}\small{n}\end{equation*} per unit time. Note that in the following, we let \begin{equation*}\small{F_{nn}=0}\end{equation*}. For what follows, only relative flux fractions

**Figure:** (1)

\begin{equation*}f_{nm}=\frac{F_{nm}}{\sum_{nm}F_{nm}}\end{equation*}

are required to specify the network. The quantities \begin{equation*}\small{f_{nm}}\end{equation*} can be interpreted as an effective coupling between districts \begin{equation*}\small{m}\end{equation*} and \begin{equation*}\small{n}\end{equation*} that is induced by the food distribution between these districts. We consider the quantities \begin{equation*}\small{f_{nm}}\end{equation*} as a proxy from which spreading propensities between \begin{equation*}\small{m}\end{equation*} and \begin{equation*}\small{n}\end{equation*} can be derived.

Because precise measurements of food distribution pathways are not available, we consider an established, approximate heuristic from the social sciences, economics and transportation theory known as the gravity model 30 ^,^ 31. This approach accounts for the observation that traffic flow increases monotonically with the population size between locations and decreases algebraically with distance, leading to the relationship

**Figure:** (2)

\begin{equation*}F_{nm} \propto \frac{N_m^\alpha  N_n^\beta}{(1 + d_{nm}/d_0)^\gamma}, \end{equation*}

where \begin{equation*}\small{N_m}\end{equation*}, \begin{equation*}\small{N_n}\end{equation*}, and \begin{equation*}\small{d_{nm}}\end{equation*} quantify the population size of origin \begin{equation*}\small{m}\end{equation*}, destination \begin{equation*}\small{n}\end{equation*}, and their geographic distance, respectively. The non-negative exponents \begin{equation*}\small{\alpha,\ \beta,\ \gamma}\end{equation*} and distance scale \begin{equation*}\small{d_0}\end{equation*} are parameters of the gravity model 32 ^,^ 33. Plausible choices for these parameters can be found in the following way: First, we assume that the coupling strength between two locations \begin{equation*}\small{m}\end{equation*} and \begin{equation*}\small{n}\end{equation*} increase with the number of connections (\begin{equation*}\small{N_n\times N_m}\end{equation*}) that can be formed between elements of the populations. This implies that \begin{equation*}\small{\alpha=\beta}\end{equation*}. Additionally, the coupling strength should be proportional to a mean value of the origin and destination population sizes, while leverage by large population nodes should be attenuated. Accounting for this, we choose the geometric average

**Figure:** (3)

\begin{equation*}F_{nm}\propto \sqrt{N_n N_m}.\end{equation*}

Furthermore, we let the coupling strength \begin{equation*}\small{F_{nm}}\end{equation*}decrease with distance. The corresponding tail exponent is consistent with the quantitative assessments of human mobility and transportation networks 34 ^,^ 35, i.e.

**Figure:** (4)

\begin{equation*}F_{nm}\propto\frac{1}{d_{nm}^{2+\mu}}\quad\text{with}\quad\mu\approx 0.6\end{equation*}

Finally, we fix the scale parameter \begin{equation*}\small{d_0}\end{equation*} (in km) in Eq. (2) to be of the order of the average linear extent of a district. With these assumptions, the parameters in the gravity model are \begin{equation*}\small{\alpha=\beta=1/2,\ \gamma=2.6}\end{equation*} and \begin{equation*}\small{d_0=10}\end{equation*} km. Although we choose these parameter values as base values, we also investigate the robustness of our results against variations in exponents and found that our results are quite robust.

The gravity model generates a fully connected network with strongly heterogeneous weights, contrasting realistic mobility or transportation networks that possess a sparse topology. In order to obtain a more realistic model for food distribution that exhibits topological sparseness of connections, we follow a procedure recently introduced by Serrano et al. 36. The idea of this approach is that only links are retained that are statistically significant with respect to a random null model, in which traffic is distributed uniformly among links of a node. Following this idea, we first compute the flux fraction

**Figure:** (5)

\begin{equation*}p_{nm}=f_{nm}/\sum_{n} f_{nm}\end{equation*}

for each node \begin{equation*}\small{m}\end{equation*}. If at each node, traffic was randomly distributed among the remaining \begin{equation*}\small{M-1}\end{equation*} other nodes, a null model would produce \begin{equation*}\small{p^0_{nm}\approx 1/M}\end{equation*}. Thus, we only retain links that possess a flux fraction larger than \begin{equation*}\small{1/M}\end{equation*}, i.e. if

**Figure:** (6)

\begin{equation*}p_{nm}>1/M.\end{equation*}

This approach yields a network skeleton of statistically significant links. Following this procedure the resulting network has an overall connectivity of 18%, see Fig. 2B.

**Multiscale Food Distribution in Germany:**
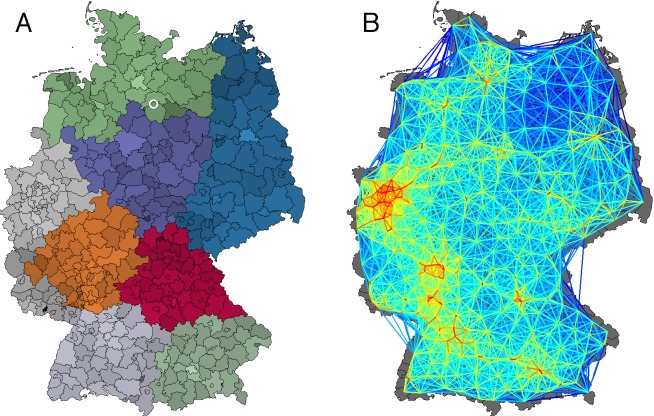
**(A)** A map of German districts; hues correspond to the regional network modules obtained by modularity maximization 37; color intensity quantifies population density. The origin of the 2011 EHEC/HUS outbreak is marked by a white circle in Bienenbüttel located in the district Uelzen. **(B)** German food shipping network constructed from a gravity model with parameters \begin{equation*}\small{\alpha=\beta=1/2,\ \gamma=2.6}\end{equation*}, and \begin{equation*}\small{d_0=10}\end{equation*} km. Each district is represented by a network node, coloring corresponds to the link strength. The network has a connectivity of 18.1%.

One of the characteristic features of transportation networks in general, which is also captured by the above gravity model, is its multiscale structure. Although short-range links are usually strongest, the algebraic tail in Eq. (2) yields long-range connections that can dominate spreading phenomena evolving on these networks. Qualitatively, this is illustrated in Fig. 3A which depicts an simple planar quasi-lattice network, in which every node is connected only to its spatially adjacent nodes. Additionally, a few long-range, random connections are added. Because of long-range connections in the network, an initially localized spreading process quickly attains a spatially incoherent structure. As a consequence of this, it is no longer possible to predict with ordinary diffusion when a spreading process will arrive at a given location in the network. More importantly, it is difficult to reconstruct the outbreak origin from a snapshot (or a sequence of snapshots) of the spatio-temporal pattern of spread alone based on conventional planar distance measures and two-dimensional geometry.

Effectively, two nodes that are connected by a long-range link in a multiscale network system are more adjacent than their spatial distance would suggest. Based on this basic and intuitive insight, a recent study 25 introduced the concept of effective distance to network-driven contagion or spreading phenomena. The most important result of this study is that spatio-temporally complex patterns of spreading can be mapped onto simple, regular wave front patterns when conventional distance is replaced by a suitably chosen effective distance. This not only permits calculations of arrival times at any node in the network but, more importantly, the identification of outbreak origins as will be explained in more detail below. The effective distance approach has been shown to work in the context of infectious disease dynamics on a global scale, for instance, the worldwide spread of SARS in 2003 and pandemic influenza H1N1 in 2009.

The effective distance method assumes that, irrespective of the details of the local dynamics of a spreading process, the proliferation of the contagion throughout the network is determined by the coupling between nodes, and that this coupling is quantified by the flux matrix elements \begin{equation*}\small{f_{nm}}\end{equation*}. Given an initial outbreak location \begin{equation*}\small{n_0}\end{equation*}, a contagion process can take a multitude of paths to any other node in the network. Each path \begin{equation*}\small{\Gamma}\end{equation*} is taken with probability \begin{equation*}\small{P(\Gamma)}\end{equation*}. Consider a path that starts at \begin{equation*}\small{n_0}\end{equation*} and ends at \begin{equation*}\small{n_K}\end{equation*} with a sequence of intermediate steps at nodes \begin{equation*}\small{n_i,\ i=1,\ldots,K-1}\end{equation*}such that

**Figure:** (7)

\begin{equation*}\Gamma = \{n_0,\ldots,n_K\}.\%0A\end{equation*}

The probability of the contagion process taking this path is assumed to be given by the product of probabilities of each step

**Figure:** (8)

\begin{equation*}P(\Gamma)=\prod_{i=1}^K P(n_i | n_{i-1}).\end{equation*}

Here, for every link in the network the function \begin{equation*}\small{P(n|m)}\end{equation*} is the probability that a contaminated food at \begin{equation*}\small{m}\end{equation*} is moved to \begin{equation*}\small{n}\end{equation*}. The fundamental assumption in Brockmann and Helbing 25 is that the single step probability \begin{equation*}\small{P(n|m)}\end{equation*} is identified with the flux fraction \begin{equation*}\small{p_{nm}}\end{equation*}that is determined by the underlying transportation network:

**Figure:** (9)

\begin{equation*}P(n|m)=p_{nm}=\frac{f_{nm}}{{\sum_{n} f_{nm}}}.\end{equation*}

Then, we define the effective distance of a multi-leg path \begin{equation*}\small{\Gamma}\end{equation*}by

**Figure:** (10)

\begin{equation*} \Lambda(\Gamma) = K -\log\left[P(\Gamma)\right], \end{equation*}

where \begin{equation*}\small{K}\end{equation*} is the number of links composing the path and \begin{equation*}\small{P(\Gamma)}\end{equation*}the corresponding path probability. For the sake of motivation and interpretation, we can decompose the path length into contributions by direct links of this formula:

**Figure:** (11)

\begin{equation*}\Lambda(\Gamma) = \sum_{i=1}^{K} \lambda(n_{i}|n_{i-1}).\end{equation*}

Here, the effective length of a direct link \begin{equation*}\small{n_{i-1}\rightarrow n_i}\end{equation*}is given by

**Figure:** (12)

\begin{equation*}\lambda(n_i|n_{i-1})=1-\log P(n_i|n_{i-1}).\end{equation*}

This relation establishes a connection between network topological features and effective distance. The functional form is chosen such that a number of important features are fulfilled: (i) the length from \begin{equation*}\small{n_{i-1}}\end{equation*} to \begin{equation*}\small{n_i}\end{equation*} decreases with increasing probability \begin{equation*}\small{P(n_i|n_{i-1})}\end{equation*}. That is, for large values of \begin{equation*}\small{P(n_i|n_{i-1})}\end{equation*}, the effective length is small and for vanishing transition probability the effective length diverges. (ii) The effective length of a multi-step path \begin{equation*}\small{\Gamma}\end{equation*} as defined in Eq. (7) is the sum of the effective lengths of each segment in the path. (iii) Given two paths that occur with certainty (e.g. with \begin{equation*}\small{P(n_i|n_{i-1})=1}\end{equation*} for each link), but have a different number of segments, the path that has more segments also has a larger effective length.

Generically, transportation networks are strongly heterogeneous such that, in an ensemble of paths with origin \begin{equation*}\small{n_0}\end{equation*} and destination \begin{equation*}\small{n_K}\end{equation*}, the dynamics are dominated by the most probable path and therefore the path of minimum effective length 25. The effective distance \begin{equation*}\small{D(n|m)}\end{equation*} is defined as the minimum effective length of a path \begin{equation*}\small{\Lambda(\Gamma)}\end{equation*} from origin \begin{equation*}\small{m}\end{equation*} to destination \begin{equation*}\small{n}\end{equation*}:

**Figure:** (13)

\begin{equation*}D(n|m)=\min\limits_{\Gamma} \Lambda(\Gamma).\end{equation*}

From the perspective of a chosen root or reference node \begin{equation*}\small{m}\end{equation*}, one can compute the shortest path tree \begin{equation*}\small{T_m}\end{equation*}, which is the collection of shortest effective paths to all other nodes in the network. This shortest path tree is equivalent to the most probable contagion hierarchy that a spreading process will take through the network.

**Effective distance and outbreak origin reconstruction in multi-scale network contagion processes.**
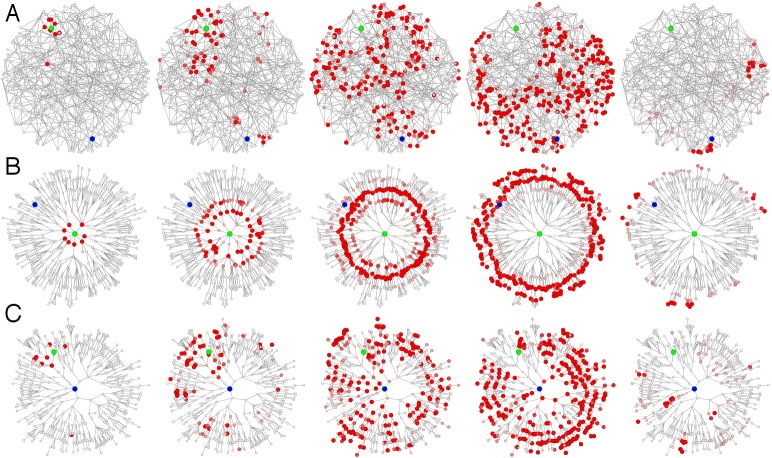
**(A)** Each panel depicts a temporal snapshot (from left to right at equidistant time intervals) in a simple contagion process in which infected nodes (red) deliver the infection to connected nodes at a fixed rate before they recover at a another rate (SIR dynamics 38). The network consists of 512 nodes on a quasi-triangular, random lattice. Each node is connected to its nearest local neighbors. In addition to the local lattice structure, 128 long range links exist between randomly chosen pairs of nodes. The origin of the outbreak is marked in green. Because of long range connectivity the pattern quickly loses spatial structure and becomes chaotic such that it is difficult to predict from metric cues alone when the contagion arrives at a given node. More importantly, long range connectivity leads to a loss of spatial coherence and it becomes impossible to determine the origin of outbreak. **(B)** The same pattern as in (A) is shown in the effective distance perspective from the outbreak origin. The depicted tree is the shortest path tree, i.e. the most probable spreading path of the contagion process. Radial distance is proportional to effective distance as defined in the text. In this alternative representation the complex pattern in the conventional view is mapped onto a simple propagating wave front and arrival times are easily computed. **(C)** The regularity of the pattern is only present from the perspective of the actual outbreak origin. When the contagion process is viewed from any other node (here the node depicted in blue), the pattern lacks regularity.

Fig. 3B illustrates the advantages of this approach in an artificial multi-scale network. From the perspective of the outbreak origin, the shortest path tree of the root node is shown, and the radial distance in the new map corresponds to the effective distance from the root node to the remaining nodes in the network. The same spreading process that appears to be spatio-temporally complex in the conventional metric layout is equivalent to a regular, constant-speed spreading wave in the effective distance representation. Consequently, one can calculate arrival times based on effective distance alone. In fact, in Brockmann and Helbing 25 it was shown that effective distance from the outbreak origin and arrival time strongly correlate in real scenarios, e.g. the 2003 SARS epidemic and the 2009 H1N1 pandemic influenza outbreak.

The most relevant consequence of the effective distance approach is that, *only* from the perspective of the actual outbreak origin, the pattern exhibits a regular concentric wave front structure. From the perspective of any other node in the network, the pattern exhibits a more or less disordered structure. Fig. 3C illustrates this. The panels depict the same dynamics as in the other panels from a randomly chosen reference node. Clearly, any spatial regularity is absent. One can now make use of this observation, i.e. the fact that the spreading pattern is regular only from the perspective of the actual outbreak location, to reconstruct the outbreak origin. Given a snapshot of the disease spread, e.g. the disease incidence at every node, one computes the effective distance perspective for each node in the network and quantifies, from which node the pattern appears to be most regular. The node with maximum regularity is considered to be the most likely outbreak origin. In the following we apply this approach to the 2011 EHEC/HUS outbreak in Germany.

**Shortest path trees and effective distance among districts in Germany.**
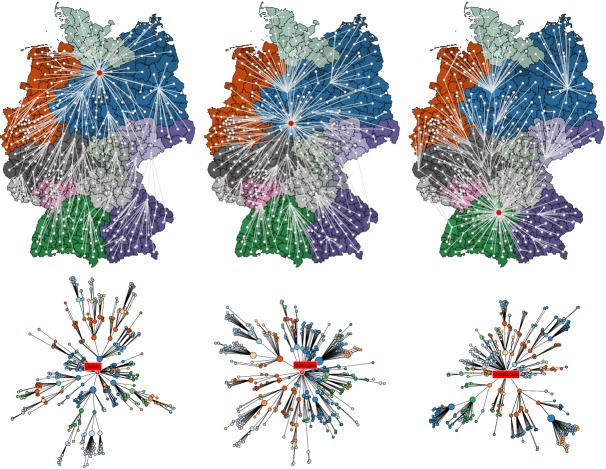
Each column depicts the shortest path tree \begin{equation*}\small{T_m}\end{equation*} for a sample root node (red), from left to right districts Uelzen, Göttingen, and Oberalbkreis. The top row depicts embedded in the conventional geographic representation, the bottom illustrates the shortest path tree in a layout such that the radial distance is proportional to the effective distance from the root node in the same way as in Fig. 3. The shortest path tree \begin{equation*}\small{T_m}\end{equation*} represents the most probable path that a contagion process takes with initial outbreak in node \begin{equation*}\small{m}\end{equation*}.

## Detection of the German EHEC/HUS outbreak origin

Given the gravity model network for food transportation, we first compute the shortest path tree \begin{equation*}\small{T_m}\end{equation*} for every potential root node \begin{equation*}\small{m}\end{equation*}, see Fig. 4 for examples. Next, a temporal snapshot of the EHEC incidence pattern is analyzed in each of the shortest path tree representations, i.e. from the perspective of all network nodes as potential candidate origins of outbreak. The incidence pattern typically consists of a subset \begin{equation*}\small{\Omega}\end{equation*} of nodes with non-zero incidence. From the perspective of the actual outbreak origin, the effective distance to these affected nodes, should be small and exhibit a small variance, a consequence of the concentricity of the spreading pattern in the effective distance representation. Therefore, in order to quantify the regularity of the incidence pattern from every potential outbreak origin, we compute the average \begin{equation*}\small{\mu(D;m)}\end{equation*} and standard deviation \begin{equation*}\small{\sigma(D;m)}\end{equation*} of effective distances to nodes with nonzero incidence (the subset of nodes \begin{equation*}\small{\Omega}\end{equation*}) 25.

**Figure:** (14)

\begin{equation*}\mu(D;m)=\frac{1}{N_\Omega}\sum_{n\in \Omega} D(n|m),\;\; \sigma^2(D;m)=\frac{1}{N_\Omega}\sum_{n\in \Omega} D(n|m)^2- \mu(D;m)^2\end{equation*}

In combination, small mean and variance are equivalent to high concentricity and, thus, high likelihood that the chosen reference node is the likely outbreak origin.

**EHEC/HUS outbreak origin reconstruction:**
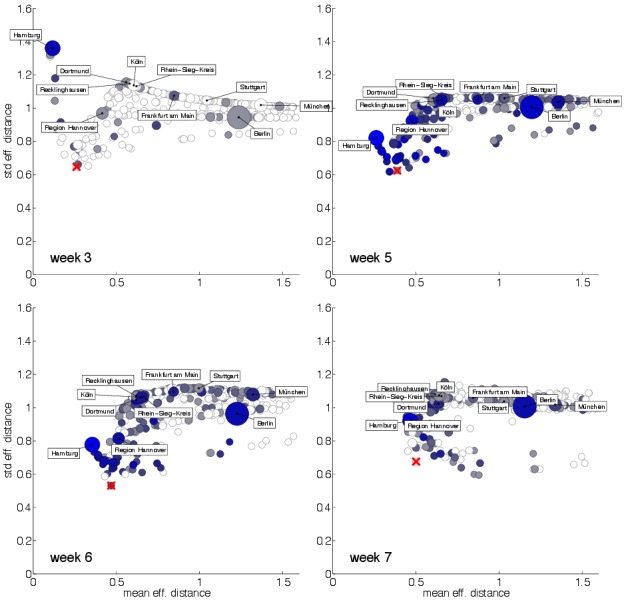
Each panel depicts a scatterplot of mean \begin{equation*}\small{\mu(D,m)}\end{equation*} and standard deviation \begin{equation*}\small{\sigma(D,m)}\end{equation*} (see Eqs. (14)) of effective distances from candidate nodes \begin{equation*}\small{m}\end{equation*} to the subset \begin{equation*}\small{\Omega}\end{equation*} of nodes that have nonzero incidence for weeks \begin{equation*}\small{t=3,\ 5,\ 6,\ 7}\end{equation*}after outbreak onset. All districts are considered as potential candidates as outbreak origin. Symbol size quantifies population size of each district, blueness quantifies incidence in the respective week. A few large district are labeled. The district with combined minimal mean and variance (closest to the origin) has a high likelihood of being the actual 2011 EHEC/HUS outbreak origin. The actual outbreak origin Uelzen in marked by a red cross.

We used the public available *E. coli* case count data with report date between calendar weeks 18 and 26 of 2011 28. According to the Task Force EHEC, this corresponds to the entire outbreak duration from May 2nd until July 4th, 2011 26. Fig. 5 shows the results of origin detection when the effective distance approach in combination with a gravity model for food distribution is applied to the EHEC incidence data. Since an *E. coli* infection clustering was noticed at May 19th, 2011 (outbreak week 3), we computed the mean \begin{equation*}\small{\mu(D,m)}\end{equation*} and standard deviation \begin{equation*}\small{\sigma(D,m)}\end{equation*} pair for weeks \begin{equation*}\small{t=3,\ 5,\ 6,\ 7}\end{equation*} and every node in the network treating every node \begin{equation*}\small{m}\end{equation*} as a potential outbreak origin. When both quantities are small, the resulting spreading patterns is most concentric in the effective distance perspective. Fig. 5 shows that already in week 3 of the event, district Uelzen is identified as the potential origin of the outbreak, this is also true for weeks 6 and 7. In week 5 the method incorrectly identifies district Lüneburg as the likely outbreak origin and Uelzen ranks third in the epicenter reconstruction. Note that the geographic center of district Lüneburg is as close to Bienenbüttel (the alleged location of contaminated sprouts) as the geographic center of Uelzen (ca. 20km). Note also, that the overall distribution of pairs \begin{equation*}\small{(\mu(D,m),\ \sigma(D,m))}\end{equation*} differs considerably for each temporal snapshot of EHEC incidence districts close to the actual outbreak location exhibit combined small values of \begin{equation*}\small{(\mu(D,m),\ \sigma(D,m))}\end{equation*}. Table in Fig. 6 ranks the candidate outbreak locations for weeks 2 to 8. The ranks were computed by comparing the effective distance to the origin in the \begin{equation*}\small{(\mu(D,m),\ \sigma(D,m))}\end{equation*} scatter plot. For all time windows except weeks 4 and 8 the correct district ranks among the top candidates for EHEC outbreak origin. Note that other potential outbreak origins are typically districts that are in close geographic proximity to the actual outbreak location. This implies that even if the origin cannot be identified on the scale of a single district, potential candidates according to the effective distance methods are confined to a small region in the vicinity of the actual outbreak location, for instance the set of neighboring districts.

**EHEC/HUS outbreak origin reconstruction:**
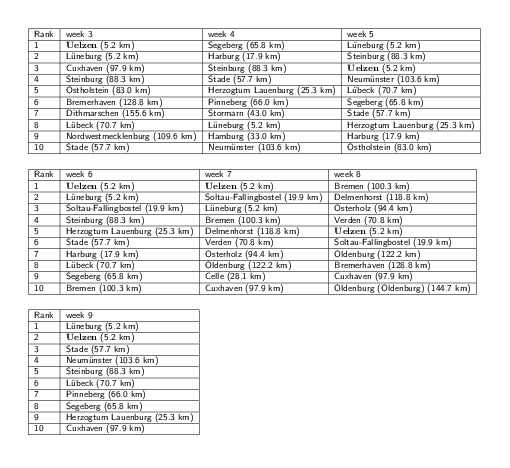
For each week 2-9 relative to the beginning of the EHEC/HUS outbreak and for each node \begin{equation*}\small{m}\end{equation*} in the network a rank was computed based on minimization of a concentricity score\begin{equation*}\small{q(m)=\sqrt{\mu^2(D,m)+\sigma^2(D,m)}}\end{equation*}. District Uelzen, the actual outbreak district is robustly ranked among the top ranked districts, in weeks 3, 6 and 7, Uelzen is ranked first. We considered all 412 districts. For each district the distance provided in parenthesis represents the approximate distance to the actual outbreak location Bienenbüttel in district Uelzen.

**Correlation of effective distance and arrival time during the German EHEC/HUS outbreak, 2011.**
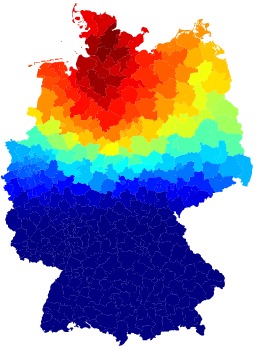
For each district as a potential outbreak origin, we computed the correlation coefficient of arrival time \begin{equation*}\small{T(n)}\end{equation*} at every other node \begin{equation*}\small{n}\end{equation*} and effective distance from \begin{equation*}\small{m}\end{equation*} to \begin{equation*}\small{n}\end{equation*}. The magnitude of the correlation coefficient is color-coded from blue to red, corresponding to low and high correlation, respectively. High correlation, corresponding to high likelihood of being the outbreak origin is observed in a spatially coherent region in Northern Germany.

The effective distance method provides an alternative method for outbreak origin reconstruction. An important result presented in Ref. 25 is that arrival times of a network-driven contagion process correlate strongly with effective distance. In fact, the arrival time \begin{equation*}\small{T_n}\end{equation*} of the process at a node \begin{equation*}\small{n}\end{equation*} with initial outbreak at node \begin{equation*}\small{m}\end{equation*} increases linearly with effective distance \begin{equation*}\small{D(n|m)}\end{equation*}. Again, arrival time and effective distance only correlate strongly when the actual outbreak origin is chosen as the reference node. To supplement the above analysis we computed the correlation coefficient \begin{equation*}\small{c(m)}\end{equation*} of arrival times (i.e. the week of reported first case of EHEC/HUS in a given district) with effective distance, considering each node \begin{equation*}\small{m}\end{equation*} of the 412 districts as the potential outbreak origin. We then ranked these correlation coefficients. Fig. 7 depicts the magnitude of \begin{equation*}\small{c(m)}\end{equation*} in a map of all German districts. Clearly, this method identifies a well-defined region in Northern Germany as containing the likely outbreak location. Note that, in contrast to the incidence patterns, the correlation coefficient varies smoothly with distance from the epicenter somewhere in Northern Germany. When correlation coefficients are ranked according to magnitude, the correct origin district Uelzen only ranks 30 out of 412 districts. However, the difference in correlation coefficients is small among the top-ranked districts, see Table in Fig. 8. The reason for the comparatively low performance of the correlation-based outbreak reconstruction could be that the temporal resolution of the data is too coarse and fluctuations dominate the signal. For instance, travel-related cases could warp the infection pattern. We conclude that outbreak origin reconstruction based on the topological features of the wave front in effective distance, as presented in Fig. 5 and Table in Fig. 6, is a more reliable technique for the detection of the outbreak origin than the correlation approach. Also, for the topological rather than the correlation-based approach only single temporal snapshots of incidence are required, which is an additional advantage.

**Effective distance and arrival time analysis:**
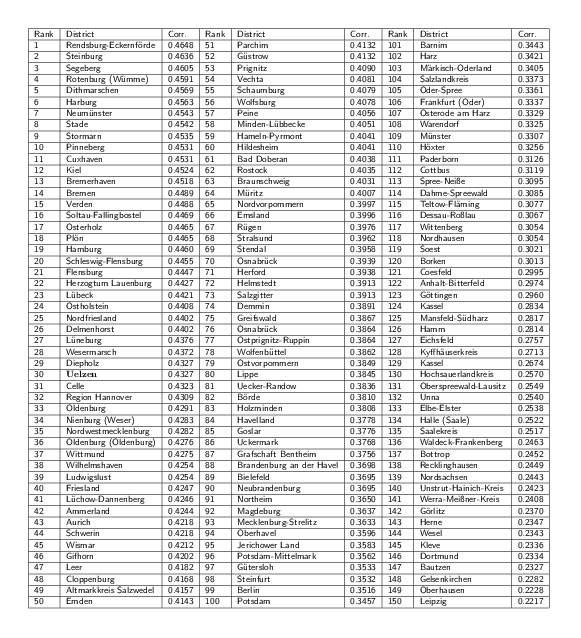
For each potential district \begin{equation*}\small{m}\end{equation*} as outbreak origin we computed the Pearson correlation of arrival time \begin{equation*}\small{T(n)}\end{equation*} and effective distance \begin{equation*}\small{D(n|m)}\end{equation*} and ranked all districts with respect to correlation magnitude. The actual outbreak origin Uelzen is ranked at position 30. High correlation districts all lie in Northern Germany.

## Discussion and conclusion

We introduced a fast and efficient approach for the identification of the origin during food-borne disease outbreaks and evaluated the approach in the context of the 2011 EHEC/HUS outbreak in Germany. A clear advantage of the method is the robust performance on the basis of limited case report data and plausible topological assumptions concerning the underlying food distribution network. When applied to the 2011 EHEC/HUS outbreak in Germany, our method was able to identify an outbreak origin in close proximity to the actual outbreak location (Uelzen, Lower Saxony). Already three days (May 22nd, 2011) after spatial infection clustering, the effective distance approach was able to reconstruct the actual origin. This is particularly promising, as in the context of EHEC/HUS, conventional outbreak investigations, including case-control- and cohort-studies as well as sample testings and tracings along the food-shipping chain,wrongly suggested tomatoes, leafy salads and cucumbers as contaminated foods. When specific suspicions arose that cucumbers imported in Hamburg would be the infection source, our method classifies Hamburg to be a very unlikely origin. The consideration of such contradictory information could have lead to more spatially targeted sample testing, and, therefore could have improved the efficiency of the outbreak investigations.

We believe that this method can complement conventional methods of origin localization of food-borne diseases and consequently facilitate a more timely success which is vital for the development of containment strategies. The underlying network definition by the gravity model is very flexible, so that the transmission vehicle does not has to be known. Basically, the network could also capture a combination of food transportation routes as well as human mobility pattern. As our method is structurally quite general and just derived from topological features of the underlying distribution networks, we believe that our approach may be adapted and applied to a variety of contagion phenomena, human-to-human transmissible diseases, and disease dynamics on individual based contact networks and human-mediated bioinvasion processes.
